# The rational application of liquid biopsy based on next‐generation sequencing in advanced non‐small cell lung cancer

**DOI:** 10.1002/cam4.5410

**Published:** 2022-11-07

**Authors:** Chenglong Zhao, Jianghua Li, Yongchang Zhang, Rui Han, Yubo Wang, Li Li, Yimin Zhang, Mengxiao Zhu, Jie Zheng, Haiwei Du, Chen Hu, Chengzhi Zhou, Nong Yang, Shangli Cai, Yong He

**Affiliations:** ^1^ Department of Respiratory Disease Daping Hospital, Army Medical University Chongqing China; ^2^ Department of Intensive care unit Daping Hospital, Army Medical University Chongqing China; ^3^ Department of Medical Oncology, Lung Cancer and Gastrointestinal Unit Hunan Cancer Hospital/The Affiliated Cancer Hospital of Xiangya School of Medicine, Central South University Changsha China; ^4^ Burning Rock Biotech Guangzhou China; ^5^ Respiratory Medicine Department, State Key Laboratory of Respiratory Disease, National Clinical Research Center for Respiratory Disease, Guangzhou Institute of Respiratory Health The First Affiliated Hospital of Guangzhou Medical University Guangzhou China

**Keywords:** circulating tumor DNA, liquid biopsy, next generation sequencing, non‐small cell lung cancer, serum tumor marker

## Abstract

**Background:**

Plasma and tissue biopsy have both used for targeting actionable driver gene mutations in lung cancer, whose concordance is imperfect. A reliable method to predict the concordance is urgently needed to ease clinical application.

**Methods:**

A total of 1012 plasma samples, including 519 with paired‐tissue biopsy samples, derived from lung adenocarcinoma patients were retrospectively enrolled. We assessed the associations of several clinicopathological characteristics and serum tumor markers with the concordance between plasma and tissue biopsies.

**Results:**

When carcinoembryonic antigen (CEA) levels were higher than thresholds of 15.01 ng/ml and 51.15 ng/ml, the positive predictive value of concordance reached 90% and 95%, respectively. When CEA levels were lower than thresholds of 5.19 ng/ml and 3.26 ng/mL, the negative predictive value of concordance reached 45% and 50%. The performance of CYFRA21‐1 in predicting concordance was similar but inferior to CEA (AUC: 0.727 vs. 0.741, *p* = 0.633). The performance of CEA combined with CYFRA21‐1 in predicting the concordance was similar to that of the combination of independent factors derived from the LASSO regression model (AUC: 0.796 vs. 0.818, *p* = 0.067). CEA (*r* = 0.47, *p* < 0.01) and CYFRA21‐1 levels (*r* = 0.45, *p* < 0.05) were significantly correlated with the maximum variant allele frequency, respectively.

**Conclusions:**

CEA combined with CYFRA21‐1 could effectively predict the concordance between plasma and tissue biopsies, which could be used for evaluating the priority of plasma and tissue biopsies for gene testing to timely guide clinical applications in advanced lung adenocarcinoma patients.

## Graphical abstract

The aim of this research was to explore a feasible and reliable method for clinicians to quickly and accurately identify patients who are suitable for plasma biopsy for genotyping first. We enrolled a total of 1012 plasma samples, including 519 with paired‐tissue biopsy samples derived from lung adenocarcinoma patients. Our findings indicated that CEA combined with CYFRA21‐1 could effectively predict the concordance between plasma and tissue biopsies based on next generation sequencing, which could be used for evaluating the priority of plasma and tissue biopsies for gene testing.

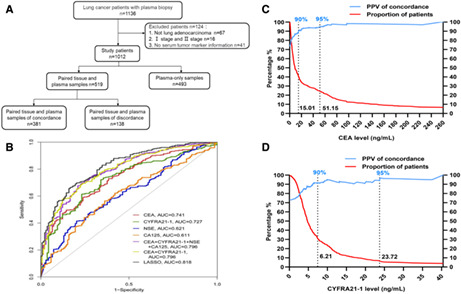

## 1 INTRODUCTION

Genetic profiling of tumors in patients with lung cancer provides the guidance of precise targeted therapy.^1^, ^2^ Tissue biopsy is the gold standard for detecting tumor genetic alterations, yet its applications are quite limited in clinical practice, due to insufficient tissue samples for testing, unreachable lesions by conventional tumor biopsy, existing contraindications to tissue biopsy, etc.^3^, ^4^ Previous studies reported that about 30% of tumor biopsy for next‐generation sequencing (NGS) analyses were failed due to insufficient tissue.^5^ Liquid biopsy, such as plasma‐based biopsy, can be an indispensable complementary modality to tissue biopsy via detecting genetic alterations of ctDNA (circulating tumor DNA), because of its advantages in safety, sparing unnecessary side effects, convenience, repeated sampling, and overcoming tumor heterogeneity.^6^ Although, the FDA has already approved the plasma test for the detection of EGFR mutations in lung cancer using PCR or NGS, the plasma‐based NGS in detecting driver gene alterations had imperfect concordance rates of about 70.0%–80.0% with tissue‐based NGS in non‐small cell lung cancer (NSCLC) patients,^7^, ^8^, ^9^, ^10^, ^11^, ^12^ which might affect the utility of plasma genotyping. Therefore, there is an urgent need to develop a reliable and convenient tool to predict the concordance between plasma and tissue biopsies to guide the appropriate utility of liquid biopsy for genotyping in advanced lung adenocarcinoma patients.

Previous studies showed that serum tumor markers, such as carcinoembryonic antigen (CEA), neuron‐specific enolase (NSE), carbohydrate antigen (CA) 19‐9, CA242, and cytokeratin fragment antigen 21‐1 (CYFRA21‐1), expressed in normal cells are abundantly synthesized and secreted in tumor cells.^13^ The levels of those serum tumor markers are frequently used to diagnose lung cancer, monitor therapeutic effects, and predict prognosis in clinical practice.^13^, ^14^ In addition, the levels of serum tumor markers were reported to be related to the presence of driver gene mutations in tumor tissues^15^, ^16^, ^17^, ^18^, ^19^, ^20^ and reflect the tumor burden of patients.^7^, ^13^ However, there are few studies on the correlation between serum tumor markers and the concordance of plasma and tissue biopsies in detecting driver genes.

Herein, we evaluated the predictive value of serum tumor markers for the concordance between plasma and tissue biopsies in detecting driver mutations in advanced lung adenocarcinoma patients and constructed a predictive model for the concordance. We aimed to provide a feasible tool to accurately identify concordance rate between plasma and tissue biopsy based on NGS in lung adenocarcinoma patients based on serum tumor markers. For the patients who present higher concordance rate, the application of plasma biopsy could be prior to tissue biopsy and therefore avoid the operative risk of tissue biopsy. What is more, for the population with lower concordance rate, tissue biopsy is more recommendable than plasma biopsy, in order to reduce the economic burden caused by unnecessary plasma biopsy.

## 2 MATERIALS AND METHODS

### 2.1 Patients

In total, 1012 patients diagnosed with stage III or IV lung adenocarcinoma were retrospectively enrolled at Chongqing Daping Hospital, Hunan Cancer Hospital, and Guangzhou First People's Hospital in China (Figure S1). These patients underwent plasma biopsy for NGS between December 2015 and March 2021. Of them, 519 patients voluntarily underwent paired plasma and tissue biopsies at the same time.^12^, ^21^ All 1012 patients were tested for serum tumor markers within 1 week of plasma biopsy and before a new round of treatment, including CEA, NSE, CYFRA21‐1, and CA125. The clinical characteristics of patients including gender, age, smoking history, and treatment status were collected according to medical records system of the hospital. Clinical staging was based on the eighth edition of the TNM classification of lung cancer. This study followed the Standards for Reporting Diagnostic Accuracy (STARD) reporting guideline and was approved by the Research Ethics Committee of Daping Hospital. This study includes no data deposited in external repositories.

### 2.2 DNA extraction and capture‐based targeted DNA sequencing

Tissue DNA and Cell‐free DNA (cfDNA) were extracted using QIAamp DNA FFPE Tissue Kit (Qiagen) at the same time. Capture‐based targeted sequencing was performed on tumor and plasma samples from 3 hospitals using a cancer gene panel spanning 237Kb of the human genome (Burning Rock Biotech), which covers the whole exons and selected introns of 168 genes, consisting of 68 lung cancer‐related genes and 100 genes related to other cancers (full description was in Supplementary Methods).

### 2.3 Normalizing the concordance between plasma and tissue biopsy genomic profiles

Concordance was defined as that a driver alteration could be detected in both plasma and tissue samples. Discordance was defined as that a driver alteration could be detected only in plasma or tissue samples.

### 2.4 Detection of serum tumor markers

Within 1 week after plasma biopsy, 3 ml of fasting venous blood was collected in the morning. Then, serum was separated for testing the levels of serum tumor markers by electrochemiluminescence immunoassay. The range of normal serum tumor markers referred to CEA 0–5.0 ng/ml, NSE 0–16.3 ng/ml, CA125 0–35 U/ml, and CYFRA21‐1 0–3.3 ng/ml.

### 2.5 Statistical analysis

All data were analyzed using the R software version 4.0.3. Continuous data conforming to the normal distribution were presented as mean ± standard deviation, and those nonconforming were presented as median (interquartile range). Categorical variables were presented as frequencies. Normality was evaluated using the Shapiro–Wilk test. In addition, Student's *t*‐test was used for normally distributed continuous variables, and the Mann–Whitney *U*‐test was performed for continuous non‐normally distributed variables. Categorical variables were analyzed using the chi‐square test. The correlation between two non‐normally distributed continuous variables was assessed by determining Spearman's rank correlation coefficient. Receiver operating characteristic (ROC) curve analyses were performed, and the method of Delong was employed to calculate the standard error of the area under the curve (AUC).^22^ The least absolute shrinkage and selection operator (LASSO) method was applied to identify independent predictors of concordance between plasma and tissue biopsies. The prediction accuracy of the nomograms was evaluated by the C‐index.^23^, ^24^ The value of the C‐index ranged from 0.0 (perfect discordance) to 1.0 (perfect concordance), and a value of 0.5 is equivalent to a random chance. Nomograms analysis was conducted for the prediction of the concordance rate between plasma and tissue samples.^24^ Bootstrapping techniques were used for internal validation of the prognostic models, and the calibration of nomograms was assessed graphically by plotting the actual probabilities versus the nomogram‐predicted probabilities.^23^, ^24^ The Cochran–Armitage trend and Wilcoxon signed‐rank test were used to performing the trend test. Values of *p* <0.05 were considered statistical significance unless otherwise specified.

## 3 RESULTS

### 3.1 Patient clinical and genetic characteristics

**FIGURE 1:**
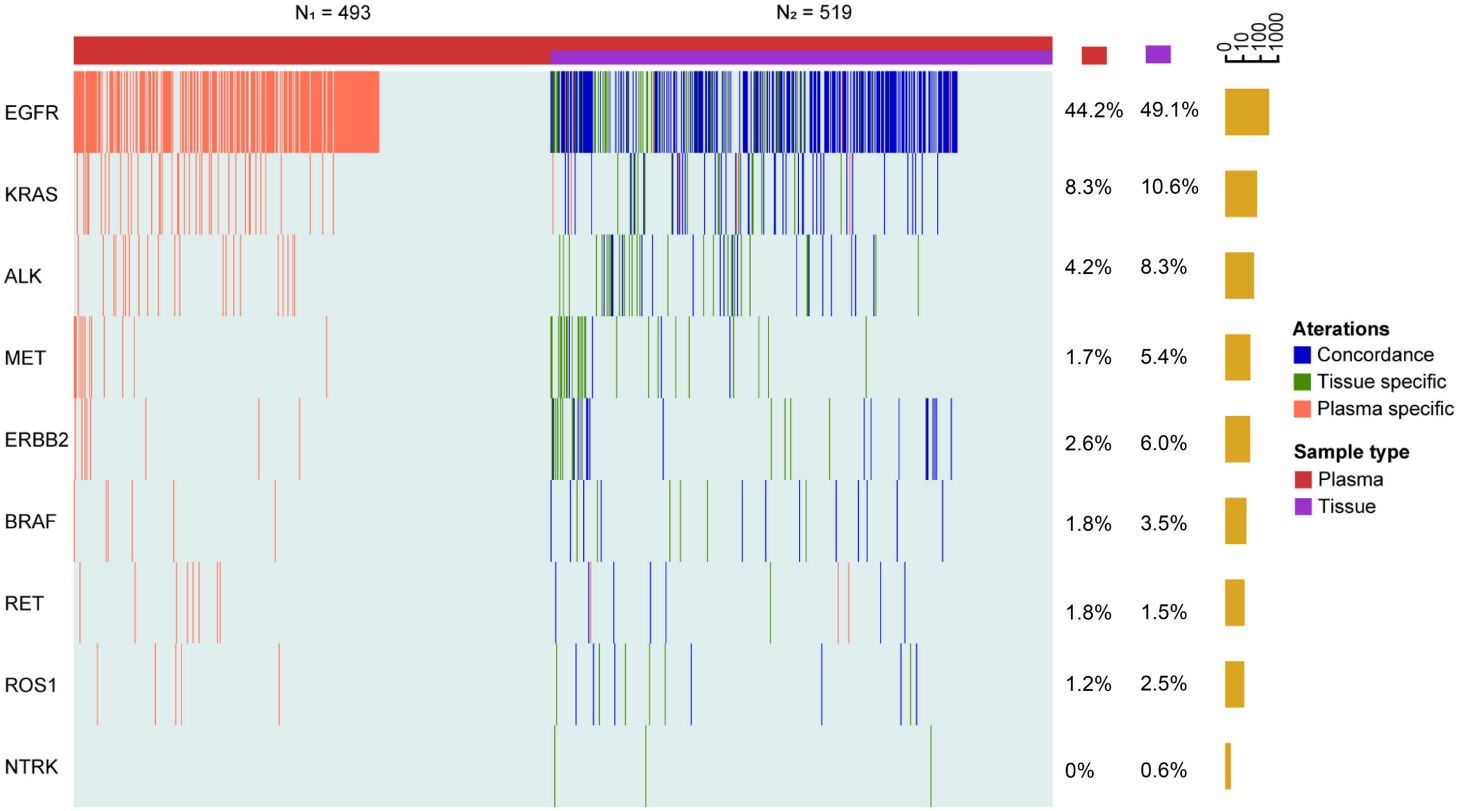
The genomic profiles in plasma and tissue samples. Comparison of mutations between plasma samples or paired tissue of each patient. N_1_ represents 493 plasma‐only samples and N_2_ represents 519 paired plasma and tissue samples. Different colors represent concordance between plasma and tissue samples, and mutations detected only in tissue samples, or only in plasma samples. Each column represents one patient. The genes are listed on the left side of the Oncoprint heat map. The mutation detection rate of plasma and tissue are displayed on right side. The far right represents the total number of each gene detected by combined plasma and tissue biopsies for all patients.

A total of 1012 patients with advanced lung adenocarcinoma were included in this study. The median age was 60 years (range 53–67) and 53.2% (538/1012) were males. The proportion of patient with distant metastases was 85.1% (861/1012). Pleura metastasis were the most frequent metastatic sites (based on the data in Table S1). Patients were divided into two groups according to the sample type: 519 patients for both plasma and tissue biopsies, and 493 patients for only plasma biopsy. Statistically significant differences between the two groups were observed in age and T stage (*p* < 0.05, Table S1), but not in sex, smoking history, distant metastasis, or clinical stages (Table S1). Nine driver genes and corresponding driver alterations recommended by the National Comprehensive Cancer Network (NCCN) guidelines with clinical therapeutic significance for lung cancer (details of driver genes are shown in Table S2) were selected to study whether the ctDNA mutational profiling based on NGS technology can accurately reflect the tumor genomic profile and overcome tumor heterogeneity.^25^, ^26^, ^27^ For plasma samples, the mutation rate of nine driver genes reached 61.9% (626/1012), and the top detected alterations were *EGFR* mutation (44.2%, 447/1012), *KRAS* mutation (8.3%, 84/1012), and *ALK* fusion (4.2%, 43/1012). For 519 tissue samples, the mutation rate of all driver genes was 79.6% (413/519). The top‐three detected alterations were consistent with plasma‐based biopsy with different ratios, that is, *EGFR* mutation (49.1%, 255/519), *KRAS* mutation (10.6%, 55/519), and *ALK* fusion (43/519, 8.3%). In addition, three cases (0.6%, 3/519) of *NTRK* fusion were detected in tissue samples but not in plasma samples (Figure 1).

### 3.2 Correlation between clinical characteristics and the concordance of plasma and paired tissue samples

**FIGURE 2:**
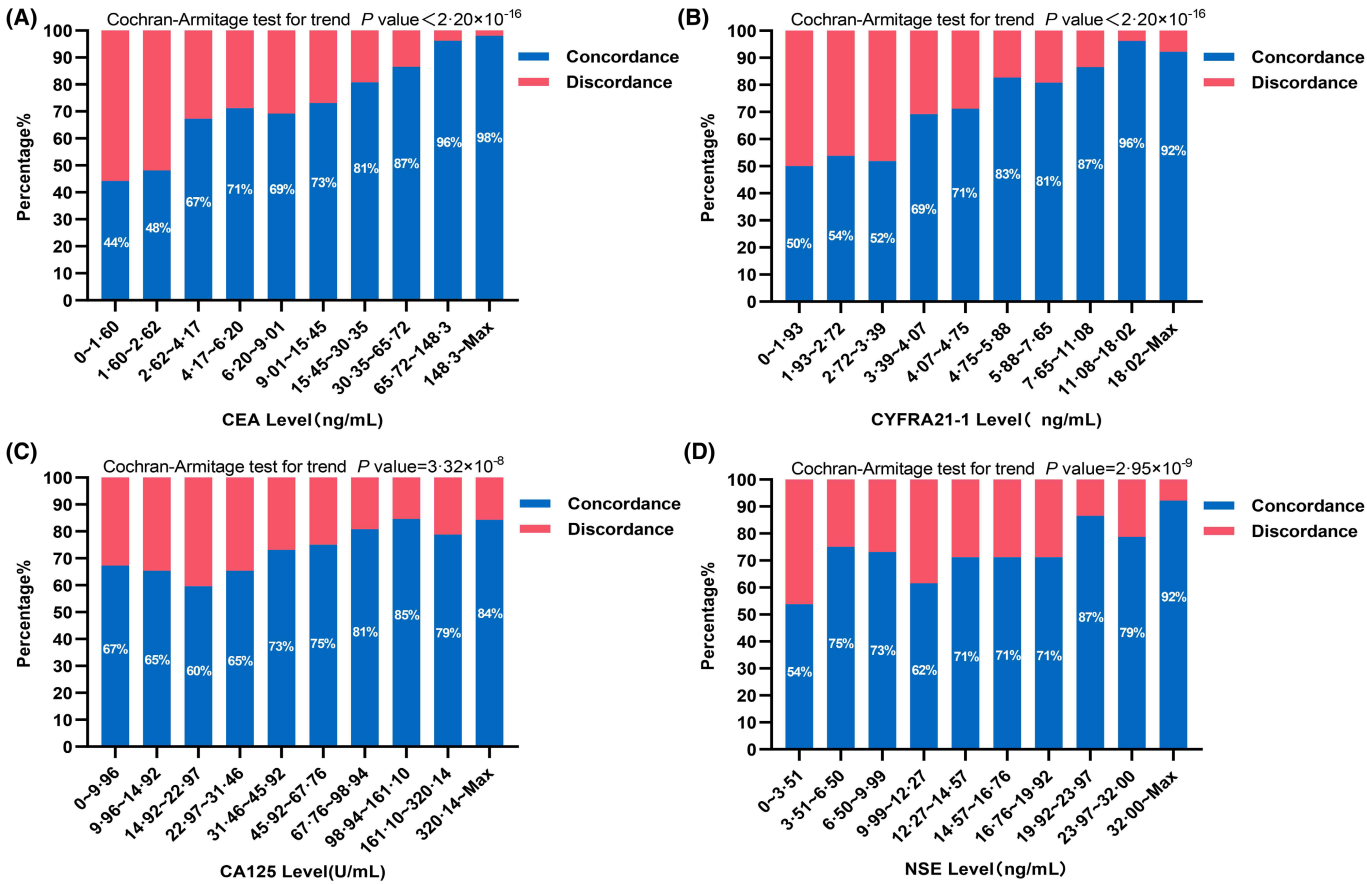
Rate of concordance between paired plasma and tissue samples at different levels of serum tumor marker. Grouping according to the decile method, and each column in histogram represents the proportion of the concordant or discordant in paired plasma and tissue samples. Cochran‐Armitage test for trends reflect the trends of concordance rate with the change levels of serum tumor markers, the smaller *p* reflects the stronger the change trend (CEA levels (A), *p* < 2.20 × 10^−16^; CYFRA21‐1 levels (B), *p* < 2.20 × 10^−16^; CA125 levels (C), *p* = 3.32 × 10^−8^; NSE levels (D), *p* = 2.95 × 10^−9^).

Of 519 patients, 381 patients had positive results of 9 driver genes in both plasma and tissue samples, reaching an overall concordance rate of 73.4% (Table 1). For the rest patients, driver gene mutation was only detected in plasma samples of 21 patients (4.1%, 21/519) (group A), of which 8 cases (38.1%, 8/21) were carrying *EGFR* T790M, and driver gene mutations were exclusively detected in tissue samples of 111 patients (111/519, 21.4%) (group B). Further univariate analysis showed that the concordance rate was significantly higher in males, M1 stage disease, and stage IV disease than in females, M0 stage disease, and stage III disease, respectively (*p* < 0.05, Table 1). Patients with bone metastases had the highest concordance rate among patients with distant metastases (*p* < 0.05, Table 1). The levels of CEA (14.68 ng/ml vs. 3.88 ng/ml, *p* < 0.001), CYFRA21‐1 (5.56 ng/ml vs. 3.25 ng/ml, *p* < 0.001), NSE (15.61 ng/ml vs. 12.03 ng/ml, *p* < 0.001), and CA125 (58.73 U/ml vs. 29.94 U/ml, *p* < 0.001) in the concordance group were significantly higher than in the discordance group (Table 1; Figure S2). Interestingly, the levels of CEA (24.50 ng/ml vs. 3.90 ng/ml, *p* < 0.001) and CYFRA21‐1 (7.22 ng/ml vs. 2.97 ng/ml, *p* < 0.001) in group A were significantly higher than in group B (Figure S3). We further conducted deciles analyses of four tumor markers in the paired samples and showed that the increased concordance rate was significantly associated with the increased level of serum tumor markers (Cochran–Armitage test for trend. CEA, *p* < 2.200 × 10^−16^; CYFRA21‐1, *p* < 2.200 × 10^−16^; CA125, *p =* 3.316 × 10^−8^; NSE, *p =* 2.951 × 10^−9^; Figure 2). Compared with CA125 and NSE, the increase trend of concordance rate in CEA and CYFRA21‐1 was more pronounced. In the subgroups of CEA ≥15.45 ng/ml and CEA ≥65.72 ng/ml, the concordance rates were more than 81% and 96%, respectively. In the subgroup of CYFRA21‐1 ≥ 5.88 ng/ml, the concordance rate was more than 81% (Figure 2).

**TABLE 1:** Characteristics of samples paired with plasma and tissue biopsies

Characteristic	Paired tissue and plasma samples (*N* = 519)	Concordance (*N* = 381)	Discordance (*N* = 138)	*p*
Age (years), median (IQR)	60.0 (51.0–66.0)	60.0 (51.0–66.0)	60.5 (51.8–66.0)	0.433
Gender, *n* (%)				0.025
Male	283 (54.5)	219 (57.5)	64 (46.4)	
Female	236 (45.5)	162 (42.5)	74 (53.6)	
Smoking status				0.153
Never	312 (60.1)	222 (58.3)	90 (65.2)	
Current or former	207 (39.9)	159 (41.7)	48 (34.8)	
T stage, *n* (%)				0.405
T1	71 (13.7)	47 (12.3)	24 (17.4)	
T2	126 (24.3)	92 (24.1)	34 (24.6)	
T3	92 (17.7)	68 (17.8)	24 (17.4)	
T4	170 (32.8)	125 (32.8)	45 (32.6)	
Unknown	60 (11.6)	49 (12.9)	11 (8.0)	
N stage, *n* (%)				0.074
N0	68 (13.1)	43 (11.3)	25 (18.1)	
N1	38 (7.3)	24 (6.2)	14 (10.1)	
N2	182 (35.1)	133 (34.9)	49 (35.5)	
N3	221 (42.6)	174 (45.7)	47 (34.1)	
Unknown	10 (1.9)	7 (1.8)	3 (2.1)	
M stage, *n* (%)				0.000
M0	88 (17.0)	48 (12.6)	40 (29.0)	
M1	431 (83.0)	333 (87.4)	98 (71.0)	
Stage, *n* (%)				0.000
III	88 (17.0)	48 (12.6)	40 (29.0)	
IV	431 (83.0)	333 (87.4)	98 (71.0)	
Metastatic site, *n* (%)				0.036
Contralateral lung	179 (34.5)	139 (36.5)	40 (29.0)	
Pleura	187 (36.0)	144 (37.8)	43 (31.2)	
Bone	188 (36.2)	167 (43.8)	21 (15.2)	
Brain	103 (19.9)	85 (22.3)	18 (13.0)	
Liver	54 (10.4)	45 (11.8)	9 (6.5)	
Pericardium	50 (9.6)	44 (11.5)	6 (4.3)	
Adrenal gland	51 (9.8)	45 (11.8)	6 (4.3)	
Others	20 (3.9)	17 (4.5)	3 (2.2)	
Therapy, *n* (%)				0.155
1st line	490 (94.0)	363 (95.3)	127 (92.0)	
≥2nd line	29 (6.0)	18 (4.7)	11 (8.0)	
CEA (ng/ml)	9.01 (3.34–47.89)	14.46 (4.79–68.13)	3.88 (1.74–8.99)	<0.001
NSE (ng/ml)	14.57 (8.21–21.67)	15.64 (8.93–22.91)	11.99 (5.02–17.54)	<0.001
CYFRA21‐1 (ng/ml)	4.75 (2.99–9.28)	5.56 (3.59–11.15)	3.25 (2.30–4.62)	<0.001
CA125 (U/ml)	45.92 (17.89–129.05)	58.68 (20.79–142.05)	29.70 (14.22–70.85)	<0.001

### 3.3 Levels of serum tumor markers predicted the concordance between paired plasma biopsy and tissue biopsy

In order to identify patients who had a high concordance or discordance rate between plasma and tissue biopsies, thresholds of serum tumor markers in patients with a high PPV (positive predictive value reflecting the concordance rate in positive serum tumor marker screening test results) or NPV (negative predictive value for the discordance rate in the negative serum tumor marker screening test results). We found that CEA thresholds were 15.01 ng/ml and 51.15 ng/ml, and CYFRA21‐1 thresholds were 6.21 ng/ml and 23.72 ng/ml in patients with the PPV of 90% and 95%, respectively (Table 2; Figure S4A,B). Furthermore, for patients with NPV of 50%, 45%, and 40%, the thresholds of CEA were 3.26 ng/ml, 5.19 ng/ml, and 7.10 ng/ml, and the CYFRA21‐1 thresholds were 2.98 ng/ml, 3.97 ng/ml, and 4.81 ng/ml, respectively (Table 2; Figure S4C,D). However, different from CEA, the NPV decreased with the decrease of CYFRA21‐1 level when it reached 50%.

**TABLE 2:** The predictive threshold value with high PPV and high NPV of concordance

Serum tumor marker	Cut‐off of concordance (%)	Threshold value (ng/ml)	Proportion of patients (%)	Sensitivity (%) (95% CI)	Specificity (%) (95% CI)
CEA	PPV				
	90.00	15.01	40.67	49.61 (44.52–54.71)	84.78 (77.73–90.35)
	95.00	51.15	24.47	31.50 (26.91–36.46)	95.65 (90.85–98.47)
	100.00	256.70	6.74	8.92 (6.30–12.20)	100.00 (97.41–100.00)
	NPV				
	40.00	7.10	44.12	63.78 (58.74–68.62)	66.67 (58.13–74.52)
	45.00	5.19	34.87	73.75 (69.05–78.13)	59.42 (50.71–67.72)
	50.00	3.26	24.08	83.73 (79.59–87.28)	45.65 (37.24–54.31)
CYFRA21‐1	PPV				
	90.00	6.21	37.76	46.19 (41.11–51.27)	86.96 (80.23–92.11)
	95.00	23.72	6.36	7.87 (5.46–11.05)	98.55 (94.91–99.79)
	100.00	40.40	3.85	4.99 (3.02–7.72)	100.00 (97.36–100.00)
	NPV				
	40.00	4.81	50.48	58.79 (53.72–63.82)	76.81 (68.92–83.64)
	45.00	3.97	38.73	70.87 (66.01–75.43)	65.94 (57.43–73.81)
	50.00	2.98	24.66	83.20 (79.15–86.85)	47.10 (38.67–55.83)

Abbreviations: NPV, negative predictive value; PPV, positive predictive value.

### 3.4 Multivariate analysis predicts concordance between plasma biopsy and tissue biopsy

**FIGURE 3:**
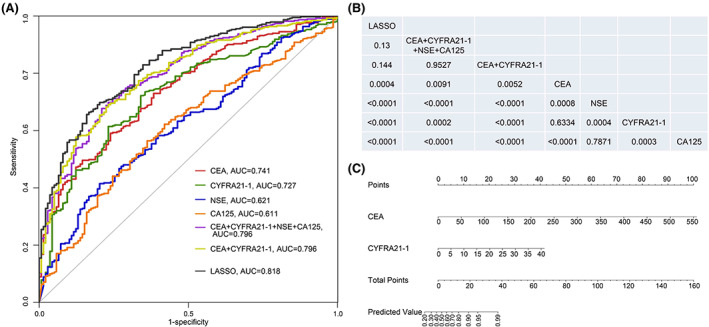
The efficacy of serum tumor markers and LASSO regression model for predicting concordance between plasma and tissue biopsies. (A) Receiver operating characteristic (ROC) curves for serum tumor markers and LASSO regression model to predict concordance; (B) Comparison of the areas under ROC Curves (AUCs) of serum tumor markers and LASSO regression model. The method of DeLong was used to compare AUCs. The figures in each column represent *p* value of difference between the factors of this column and the corresponding factors on the right columns; (C) The nomogram constructed by CEA and CYFRA21‐1 used to predict the concordance rate between plasma and tissue biopsy. Each of the variable level of serum tumor marker was given a score on the point scale axis. A total score could be easily calculated by adding each single score. By projecting the total score to the lower total point scale, we were able to estimate the probability of concordance between plasma and tissue biopsy.

The LASSO regression analysis showed that several independent factors were associated with the concordance, including male (odds ratio [OR] (95% confidence interval [CI]): 1.85 (1.18, 2.93), *p* = 0.008), M1 stage disease (OR (95% CI): 1.65 (0.94, 2.88), *p* = 0.079), bone metastasis (OR (95% CI): 3.10 (1.78, 5.57), *p* < 0.001), increased CEA level (every 10 ng/ml increment, OR (95% CI): 1.53 (1.28, 1.93), *p* < 0.001), increased CYFRA21‐1 level (every 10 ng/ml increment, OR (95% CI): 2.62 (1.55, 4.91), *p* = 0.001), increased NSE level (every 10 ng/ml increment, OR (95% CI): 1.29 (1.01, 1.66), *p* = 0.049) (Figure S5). We then analyzed the predictive efficacy of serum tumor markers and their association with multiple independent factors through ROC curves. In 519 paired plasma‐and‐tissue samples, the area under ROC curve (AUC) of CEA, CYFRA21‐1, NSE, and CA125 was 0.741, 0.727, 0.621, and 0.611, respectively (Figure 3A). The individual model of CEA or CYFRA21‐1 had a larger AUC than the other two serum tumor markers (*p* < 0.001, Figure 3B). The AUC of CEA combined with CYFRA21‐1 was equal to that of the combination of 4 serum tumor markers and smaller than that of LASSO regression model, but there were no statistical differences in the predictive efficacy among them (*p* > 0.05, Figure 3A,B). The efficacy of CEA combined with CYFRA21‐1 in predicting the concordance was higher than that of any individual serum tumor marker (*p* < 0.05, Figure 3B). Collectively, the combination of four serum tumor markers and LASSO regression model are the most efficient in predicting concordance between plasma and paired tissue samples. Next, a nomogram model based on the four serum tumor markers was constructed to predict the concordance rate between plasma and tissue samples (Figure S6), which revealed a comparable performance in predicting the concordance to CEA combined with CYFRA21‐1. Therefore, only CEA combined with CYFRA21‐1 were included in the optimized the nomogram (Figure 3C). Internal validation and decision curve analysis of clinical benefit shows that this model had clinical utility along with stable diagnostic efficiency (Figure S7).

### 3.5 Correlation analyses of serum tumor markers with mutation rate of driver genes, cfDNA concentration, and maximum variant allele frequency of ctDNA

**FIGURE 4:**
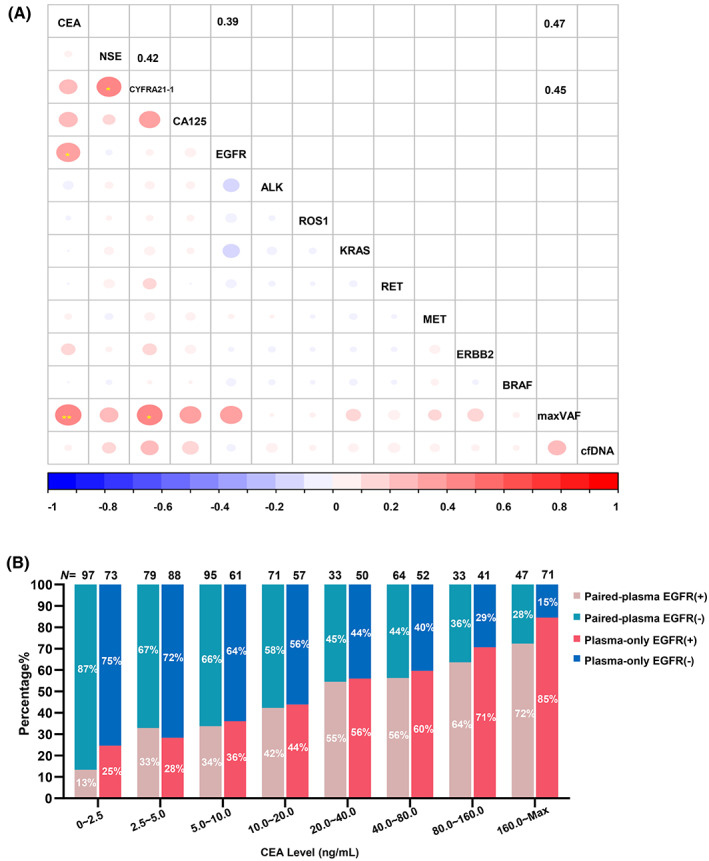
Correlation between serum tumor marker, detection rate of driver mutations and MaxVAF in plasma. (A) Correlation matrix between serum tumor markers and gene mutation profile. Correlation analysis and visualization were performed by using R package corrplot (version 0.90). *Represents that *p* value was <0.05. **Represents that *p* value was less than 0.01. (B) The paired‐plasma samples and plasma‐only samples were grouped according to equal multiples of CEA level. The proportion of EGFR mutations in each group was calculated and compared between paired‐plasma samples and plasma‐only samples by Wilcoxon signed‐rank test (*p* = 0.168). MaxVAF, maximum variant allele frequency.

CEA level was correlated with the mutation detection rate of *EGFR* in plasma (*r* = 0.39, *p* < 0.05, Figure 4A), and no more associations of driver gene mutations with serum tumor markers were observed (Figure 4A; Figure S8). Both CEA (*r* = 0.47, *p* < 0.01) and CYFRA21‐1 level (*r* = 0.45, *p* < 0.05, Figure 4A) were significantly correlated with the maximum variant allele frequency (maxVAF) of ctDNA. In addition, the AUC of maxVAF in predicting concordance was 0.836 (Figure S9). The relationship between the CEA level and mutation rate of *EGFR* in total plasma samples, paired‐plasma samples, and plasma‐only samples was subsequently explored. The CEA level was positively correlated with *EGFR* mutation rate in each group (*p* < 0.001, Figure S10A). *EGFR* mutation rate in the plasma sample of CEA‐positive patients with the CEA level >5 ng/ml was significantly higher than that in CEA‐negative patients (54% vs. 24%, *p* < 0.001, Figure S10B). Then, the CEA level was stratified in equal increasing multiples in plasma‐paired and plasma‐only sample groups, the *EGFR* mutation rate increased gradually with the increase of CEA level in both groups (*p* for trend <0.001, Figure 4B). The Wilcoxon signed‐rank test showed that the change trend of *EGFR* mutation rate was comparable between the two groups, and there was no significant difference in *EGFR* mutation rates in each same CEA range (*p* = 0.168, Figure 4B).

## 4 DISCUSSION

Tissue‐based biopsy is regarded as the gold standard for the detection of genetic alterations in clinical applications. However, the limited quantity and quality of tumor tissue hindered its clinical operations. The utility of liquid biopsy appropriately could complement the shortage of qualified tumor samples in clinic. In this study, we analyzed the correlation of four serum tumor markers and the concordance rates of plasma and tumor biopsies. The level of CEA 15.01 ng/ml or CYFRA21‐1 6.21 ng/ml could be the threshold of 90% PPV of the concordance rate, and CEA 3.26 ng/ml or CYFRA21‐1 2.98 ng/ml could be the threshold of 50% NPV of the discordance rate. We constructed a predictive model based on the level of both CEA and CYFRA21‐1 to evaluate the utility of plasma samples to diagnose genetic alterations of advanced lung cancer based on the high concordance or discordance of plasma and tumor biopsies.

In clinical applications, the tumor tissue might be unavailable due to the low compliance or the difficulties in operation. Even if the sample was obtained, the quality was another major concern. In the Checkmate 227 study, the tissue sample was collected in 94.8% (1679/1739) patients. However, only 57.7% (1004/1739) of the tumor sample was left for tumor mutational burden study.^28^ Also other studies reported that about 70% of tumor samples were qualified for NGS.^7^ With our nomogram model (Figure 3C), the clinical physicians could calculate a predictive score based on the level of CEA and CYFRA21‐1, which were part of the routine exam for lung cancer patients. For example, the total score was about 24 in a patient with CEA of 50 ng/ml and CYFRA21‐1 of 15 ng/ml, who was then predicted to have a concordance rate higher than 90% based on the nomogram model. For this patient, plasma could be used prior to tissue samples for tumor genotyping.

In this study, we first used the cancer‐gene NSG panel to analyze 9 lung cancer driver genes in 519 patients with paired plasma and tissue samples and found the concordance of plasma and tissue biopsies was 73.4%, similar to previous studies.^8^, ^9^, ^10^, ^11^, ^12^, ^29^, ^30^ We also found that the higher concordance rate of plasma and tissue biopsies were correlated with more malignant cancer stages and males. Patients with bone metastases showed the highest concordance rate in the distant metastases, implying a preference for plasma biopsy in clinic.

Serum tumor markers are abnormally expressed by malignant tumor cells, or derived from the host response to tumor, which can reflect the occurrence and development of tumor and monitor tumor response to treatment.^31^ In our study, we found that the level of serum tumor markers, CEA, CYFRA21‐1, NSE, and CA125, was all associated with the concordance rate of plasma and tissue biopsies. This funding is consistent with previous studies on the correlation between the level of CEA, CA125, and CYFRA21‐1 and the stages of lung adenocarcinoma. All three tumor markers were correlated with TNM stage of lung adenocarcinoma^32^, ^33^ and serum tumor markers are associated with tumor burden,^32^, ^33^, ^34^ implying the correction of serum tumor marker with both tissue and plasma samples.

The further exploration of the relationship between the levels of linear changes in serum tumor markers and concordance rates indicates that the concordance rate increased with the increased level of serum tumor marker, especially CEA and CYFRA21‐1. The thresholds of CEA and CYFRA21‐1 in predicting concordance rates of 90% to 95% and discordance of 40% to 50%. We found that CEA and CYFRA21‐1 threshold was 15.01 ng/ml and 7.43 ng/ml in patients having a PPV of 90%, respectively. We also found that a predictive model of CEA combined with CYFRA21‐1 outperformed individuals of CEA, CYFRA21‐1, NES, and CA125, showing similar predictive efficacy to the combination of the four tumor markers and the LASSO model. CEA combined with CYFRA21‐1 was also recommended for assisting the diagnosis of lung adenocarcinoma,^32^ while some studies reported that CEA was expressed higher in squamous cell cancer.^35^ Similarly, a previous study on paired tissue and plasma biopsy study of 185 patients showed that a scoring system based on the serum tumor markers, CYFRA21‐1, and CA19‐9 predicted more than 90% concordance between paired tissue and plasma samples,^36^ indicting the use of liquid biopsy. Although CEA was also included in this study,^36^ different experiment designs could be the main reason for the differences between two models, as the author pre‐classified lung cancer driver genes and 8 different driver genes were analyzed primarily in their study.

Furthermore, we found that the *EGFR* mutation rate in plasma increased with the increment of CEA level, consistent with previous research results in tissues.^37^, ^38^ However, no correlation was observed between other serum tumor markers and mutation rates of other driver genes. *EGFR* mutation rate in the group of plasma‐only samples was comparable with that in the group of paired‐plasma samples at the same CEA level, suggesting that CEA has similar efficacy in predicting concordance in plasma‐only and paired‐plasma samples. Therefore, patients with high levels of CEA should be recommended for detecting *EGFR* mutation in plasma.

Through a multicenter clinical study with large samples, we found that serum tumor markers, especially CEA combined with CYFRA21‐1, could effectively predict the concordance rates between plasma and tissue biopsies, and provided a predictive model of serum tumor markers for clinicians to quickly and accurately identify patients who are suitable for plasma biopsy for genotyping first. This study had some limitations. First, it was a retrospective real‐world study. Second, only four serum tumor markers, which were used to monitor diseases in the clinical diagnosis and treatment in all three hospitals, were included. Other markers associated with lung cancer were not included in this study such as tissue polypeptide antigen (TPA). Moreover, changes in the predictive efficacy of serum tumor marker during the course of treatment were not further explored. A well‐designed, prospective study is needed to stratify patients according to tumor stage, histological subtype, and treatment line to validate our findings in future work.

## 5 CONCLUSIONS

In summary, CEA combined with CYFRA21‐1 can effectively predict the concordance rate of plasma and tumor biopsies in advanced lung adenocarcinoma patients. Elevated CEA levels also could predict the presence of *EGFR* mutations in plasma. Our study provides a nomogram model based on CEA and CYFRA21‐1 as a convenient tool for individualized prediction of the concordance of plasma and tumor biopsies, to optimize the utility of plasma biopsy in genotyping for advanced lung adenocarcinoma patients.

## AUTHOR CONTRIBUTIONS

**Chenlong Zhao:** Conceptualization (equal); formal analysis (equal); methodology (equal); visualization (equal); writing – original draft (lead). **JiangHua Li:** Conceptualization (equal); formal analysis (equal); investigation (equal); software (equal); writing – original draft (equal). **Yongchang Zhang:** Data curation (equal); project administration (equal). **Rui Han:** Data curation (equal); formal analysis (equal); resources (equal). **Yubo Wang:** Data curation (equal). **Li Li:** Data curation (equal). **Yimin Zhang:** Data curation (equal); investigation (equal). **Mengxiao Zhu:** Methodology (equal); validation (equal); writing – original draft (supporting). **Jie Zheng:** Data curation (equal). **Haiwei Du:** Methodology (equal); writing – review and editing (equal). **Chen Hu:** Data curation (equal). **Nong Yang:** Data curation (equal); project administration (equal). **Chengzhi Zhou:** Data curation (equal); project administration (equal). **Shangli Cai:** Writing – review and editing (equal). **Yong He:** Conceptualization (equal); methodology (equal); project administration (equal); resources (equal); writing – review and editing (equal).

## FUNDING INFORMATION

This work was supported by the National Natural Science Foundation of China (81972189, 81802293, 82172623), the Chongqing Science Technology Commission (cstc2021jcyj‐msxmX0014), Key Technology Project for Prevention and Control of Major Diseases in Chongqing (2019ZX002), Clinical medical technology innovation ability training program (2019CXLCA003), Clinical Medical Research Talent Training Program of Army Medical University (2018XLC1015).

## CONFLICT OF INTEREST

The authors declare that they have no competing interests.

## ETHICS STATEMENT

Tissue and plasma samples of patient were obtained with informed consent, under the protocol approved by the Research Ethics Committee of Daping Hospital.

## CONSENT FOR PUBLICATION

All patients involved in our study obtained written consent for publication.

## Supporting information


Figure S1

Figure S2

Figure S3

Figure S4

Figure S5

Figure S6

Figure S7

Figure S8

Figure S9

Figure S10

Appendix S1

Table S1
Table S2

## Data Availability

All data generated or analyzed during this study are included in this published article (and its supplementary information files).

## References

[1] Hirsch FR , Scagliotti GV , Mulshine JL , et al. Lung cancer: current therapies and new targeted treatments. Lancet. 2017;389:299‐311. 27574741 10.1016/S0140-6736(16)30958-8

[2] Ettinger DS , Wood DE , Aisner DL , et al. NCCN guidelines insights: non‐small cell lung cancer, version 2.2021. J Natl Compr Canc Netw. 2021;19:254‐266. 33668021 10.6004/jnccn.2021.0013

[3] Vanderlaan PA , Yamaguchi N , Folch E , et al. Success and failure rates of tumor genotyping techniques in routine pathological samples with non‐small‐cell lung cancer. Lung Cancer. 2014;84:39‐44. 24513263 10.1016/j.lungcan.2014.01.013 PMC3954776

[4] Ignatiadis M , Lee M , Jeffrey SS . Circulating tumor cells and circulating tumor DNA: challenges and opportunities on the path to clinical utility. Clin Cancer Res. 2015;21:4786‐4800. 26527805 10.1158/1078-0432.CCR-14-1190

[5] Al‐Kateb H , Nguyen TT , Steger‐May K , Pfeifer JD . Identification of major factors associated with failed clinical molecular oncology testing performed by next generation sequencing (NGS). Mol Oncol. 2015;9:1737‐1743. 26071350 10.1016/j.molonc.2015.05.004 PMC5528718

[6] Aggarwal C , Rolfo CD , Oxnard GR , Gray JE , Sholl LM , Gandara DR . Strategies for the successful implementation of plasma‐based NSCLC genotyping in clinical practice. Nat Rev Clin Oncol. 2021;18:56‐62. 32918064 10.1038/s41571-020-0423-x

[7] Jahangiri L , Hurst T . Assessing the concordance of genomic alterations between circulating‐free DNA and tumour tissue in cancer patients. Cancers (Basel). 2019;11:11. 10.3390/cancers11121938 PMC6966532 31817150

[8] Park S , Olsen S , Ku BM , et al. High concordance of actionable genomic alterations identified between circulating tumor DNA‐based and tissue‐based next‐generation sequencing testing in advanced non‐small cell lung cancer: The Korean Lung Liquid Versus Invasive Biopsy Program. Cancer. 2021;127:3019‐3028. 33826761 10.1002/cncr.33571

[9] Leighl NB , Page RD , Raymond VM , et al. Clinical utility of comprehensive cell‐free DNA analysis to identify genomic biomarkers in patients with newly diagnosed metastatic non‐small cell lung cancer. Clin Cancer Res. 2019;25:4691‐4700. 30988079 10.1158/1078-0432.CCR-19-0624

[10] Xie FF , Zhang YJ , Mao XW , et al. Comparison of genetic profiles among primary lung tumor, metastatic lymph nodes and circulating tumor DNA in treatment‐naive advanced non‐squamous non‐small cell lung cancer patients. Lung Cancer. 2018;121:54‐60. 29858028 10.1016/j.lungcan.2018.05.002

[11] Jiang J , Adams HP , Yao L , et al. Concordance of genomic alterations by next‐generation sequencing in tumor tissue versus cell‐free DNA in stage I‐IV non‐small cell lung cancer. J Mol Diagn. 2020;22:228‐235. 31837429 10.1016/j.jmoldx.2019.10.013

[12] Aggarwal C , Thompson JC , Black TA , et al. Clinical implications of plasma‐based genotyping with the delivery of personalized therapy in metastatic non‐small cell lung cancer. JAMA Oncol. 2019;5:173‐180. 30325992 10.1001/jamaoncol.2018.4305 PMC6396811

[13] Hoseok I , Cho JY . Lung cancer biomarkers. Adv Clin Chem. 2015;72:107‐170. 26471082 10.1016/bs.acc.2015.07.003

[14] Duffy MJ , O'Byrne K . Tissue and blood biomarkers in lung cancer: a review. Adv Clin Chem. 2018;86:1‐21. 30144837 10.1016/bs.acc.2018.05.001

[15] Feng LX , Wang J , Yu Z , et al. Clinical significance of serum EGFR gene mutation and serum tumor markers in predicting tyrosine kinase inhibitor efficacy in lung adenocarcinoma. Clin Transl Oncol. 2019;21:1005‐1013. 30637712 10.1007/s12094-018-02014-6

[16] Wang S , Ma P , Ma G , et al. Value of serum tumor markers for predicting EGFR mutations and positive ALK expression in 1089 Chinese non‐small‐cell lung cancer patients: a retrospective analysis. Eur J Cancer. 2020;124:1‐14. 31707279 10.1016/j.ejca.2019.10.005

[17] Cho A , Hur J , Moon YW , et al. Correlation between EGFR gene mutation, cytologic tumor markers, 18F‐FDG uptake in non‐small cell lung cancer. BMC Cancer. 2016;16:224. 26979333 10.1186/s12885-016-2251-z PMC4793740

[18] Thomsen M , Skovlund E , Sorbye H , et al. Prognostic role of carcinoembryonic antigen and carbohydrate antigen 19‐9 in metastatic colorectal cancer: a BRAF‐mutant subset with high CA 19‐9 level and poor outcome. Br J Cancer. 2018;118:1609‐1616. 29872151 10.1038/s41416-018-0115-9 PMC6008450

[19] Oner AO , Budak ES , Yildirim S , Aydin F , Sezer C . The value of (18)FDG PET/CT parameters, hematological parameters and tumor markers in predicting KRAS oncogene mutation in colorectal cancer. Hell J Nucl Med. 2017;20:160‐165. 10.1967/s002449910557 28697193

[20] Tsang JY , Kwok YK , Chan KW , et al. Expression and clinical significance of carcinoembryonic antigen‐related cell adhesion molecule 6 in breast cancers. Breast Cancer Res Treat. 2013;142:311‐322. 24186057 10.1007/s10549-013-2756-y

[21] Vaclova T , Grazini U , Ward L , et al. Clinical impact of subclonal EGFR T790M mutations in advanced‐stage EGFR‐mutant non‐small‐cell lung cancers. Nat Commun. 2021;12:1780. 33741979 10.1038/s41467-021-22057-8 PMC7979775

[22] DeLong ER , DeLong DM , Clarke‐Pearson DL . Comparing the areas under two or more correlated receiver operating characteristic curves: a nonparametric approach. Biometrics. 1988;44:837‐845. 3203132

[23] Balachandran VP , Gonen M , Smith JJ , DeMatteo RP . Nomograms in oncology: more than meets the eye. Lancet Oncol. 2015;16:e173‐e180. 25846097 10.1016/S1470-2045(14)71116-7 PMC4465353

[24] Iasonos A , Schrag D , Raj GV , Panageas KS . How to build and interpret a nomogram for cancer prognosis. J Clin Oncol. 2008;26:1364‐1370. 18323559 10.1200/JCO.2007.12.9791

[25] Lanman RB , Mortimer SA , Zill OA , et al. Analytical and clinical validation of a digital sequencing panel for quantitative, highly accurate evaluation of cell‐free circulating tumor DNA. PLoS ONE. 2015;10:e0140712. 26474073 10.1371/journal.pone.0140712 PMC4608804

[26] Paweletz CPL , Christie J , Oxnard GR . Does testing error underlie liquid biopsy discordance? JCO precision. Oncology. 2019;3:1‐3. 10.1200/PO.18.00408 35100674

[27] Bettegowda C , Sausen M , Leary RJ , et al. Detection of circulating tumor DNA in early‐ and late‐stage human malignancies. Sci Transl Med. 2014;6:224ra224. 10.1126/scitranslmed.3007094 PMC4017867 24553385

[28] Hellmann MD , Ciuleanu TE , Pluzanski A , et al. Nivolumab plus ipilimumab in lung cancer with a high tumor mutational burden. N Engl J Med. 2018;378:2093‐2104. 29658845 10.1056/NEJMoa1801946 PMC7193684

[29] Wen S , Dai L , Wang L , et al. Genomic signature of driver genes identified by target next‐generation sequencing in Chinese non‐small cell lung cancer. Oncologist. 2019;24:e1070‐e1081. 30902917 10.1634/theoncologist.2018-0572 PMC6853120

[30] Fu Y , Wang A , Zhou J , et al. Advanced NSCLC patients with EGFR T790M harboring TP53 R273C or KRAS G12V cannot benefit from Osimertinib based on a clinical multicentre study by tissue and liquid biopsy. Front Oncol. 2021;11:621992. 33718183 10.3389/fonc.2021.621992 PMC7943858

[31] Nakamura H , Nishimura T . History, molecular features, and clinical importance of conventional serum biomarkers in lung cancer. Surg Today. 2017;47:1037‐1059. 28229299 10.1007/s00595-017-1477-y

[32] Chen ZQ , Huang LS , Zhu B . Assessment of seven clinical tumor markers in diagnosis of non‐small‐cell lung cancer. Dis Markers. 2018;2018:9845123‐9845127. 30647803 10.1155/2018/9845123 PMC6311751

[33] Chen Z , Wang Y , Fang M . Analysis of tumor markers in pleural effusion and serum to verify the correlations between serum tumor markers and tumor size, TNM stage of lung adenocarcinoma. Cancer Med. 2020;9:1392‐1399. 31881123 10.1002/cam4.2809 PMC7013070

[34] Cedres S , Nunez I , Longo M , et al. Serum tumor markers CEA, CYFRA21‐1, and CA‐125 are associated with worse prognosis in advanced non‐small‐cell lung cancer (NSCLC). Clin Lung Cancer. 2011;12:172‐179. 21663860 10.1016/j.cllc.2011.03.019

[35] Okada M , Nishio W , Sakamoto T , et al. Effect of histologic type and smoking status on interpretation of serum carcinoembryonic antigen value in non‐small cell lung carcinoma. Ann Thorac Surg. 2004;78:1004‐1009. discussion 1009–1010. 15337038 10.1016/j.athoracsur.2004.03.019

[36] Jiao XD , Ding LR , Zhang CT , et al. Serum tumor markers for the prediction of concordance between genomic profiles from liquid and tissue biopsy in patients with advanced lung adenocarcinoma. Transl Lung Cancer Res. 2021;10:3236‐3250. 34430361 10.21037/tlcr-21-543 PMC8350084

[37] Jin B , Dong Y , Wang HM , Huang JS , Han BH . Correlation between serum CEA levels and EGFR mutations in Chinese nonsmokers with lung adenocarcinoma. Acta Pharmacol Sin. 2014;35:373‐380. 24487967 10.1038/aps.2013.164 PMC4647899

[38] Gao Y , Song P , Li H , Jia H , Zhang B . Elevated serum CEA levels are associated with the explosive progression of lung adenocarcinoma harboring EGFR mutations. BMC Cancer. 2017;17:484. 28705152 10.1186/s12885-017-3474-3 PMC5512835

