# Supplementary figures and images for: The rational application of liquid biopsy based on next‐generation sequencing in advanced non‐small cell lung cancer
Source: Cancer Med. 2022 Nov 7;12(5):5603–14. doi: 10.1002/cam4.5410 (PMC10028052; doi:10.1002/cam4.5410)

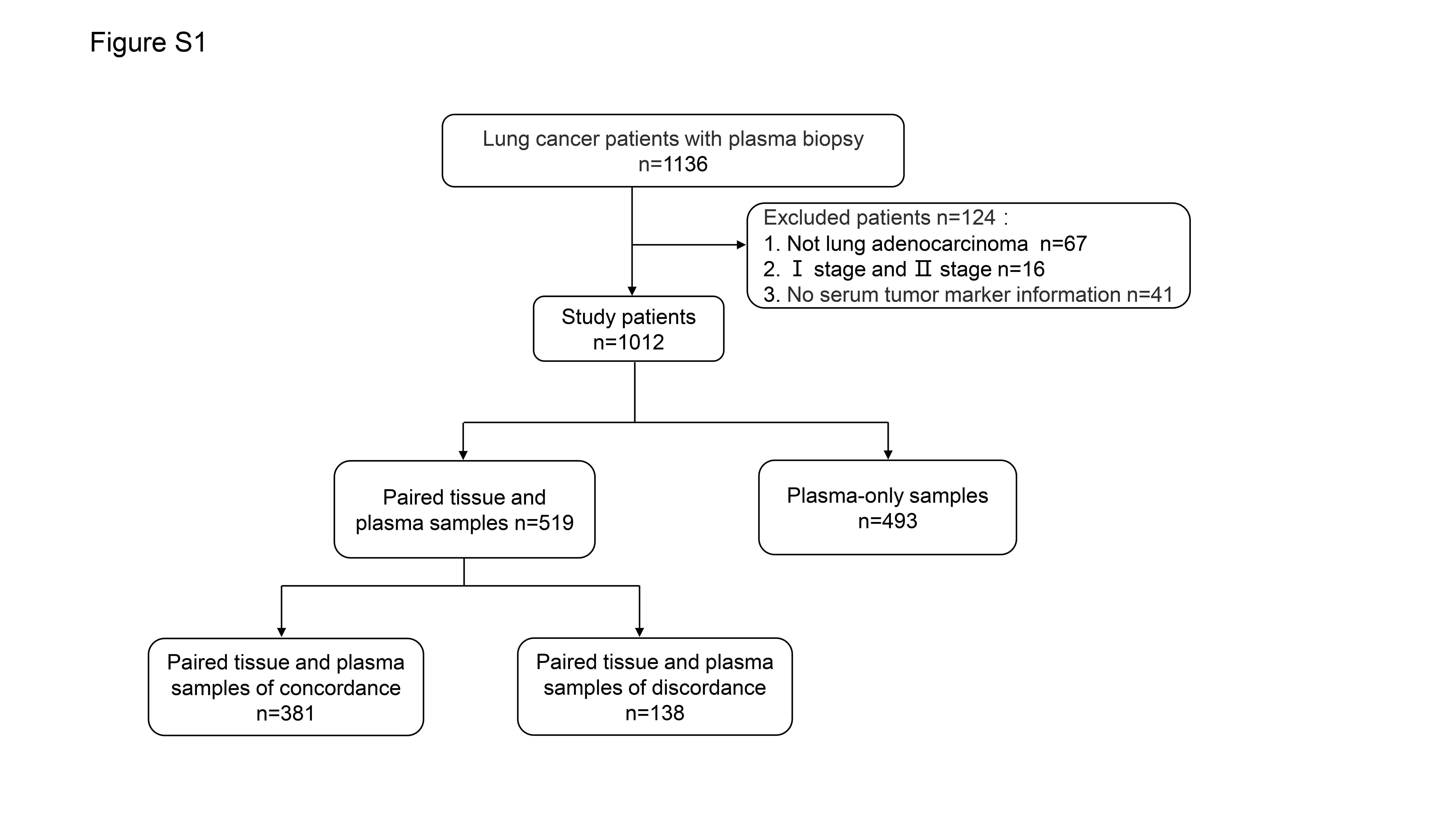

Supplement: Supplementary file 1 — Figure S1 [file CAM4-12-5603-s002.jpg]

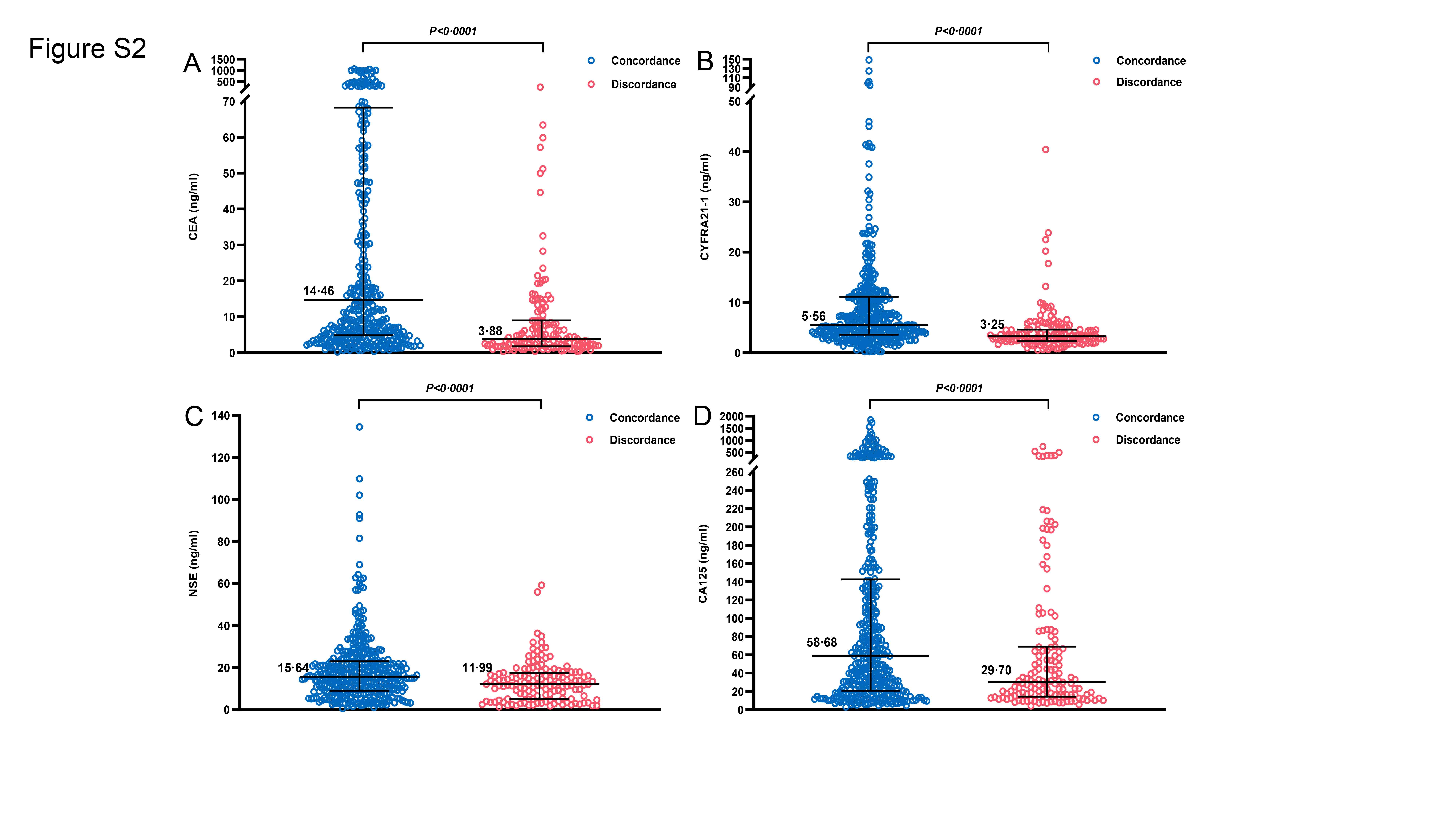

Supplement: Supplementary file 2 — Figure S2 [file CAM4-12-5603-s009.jpg]

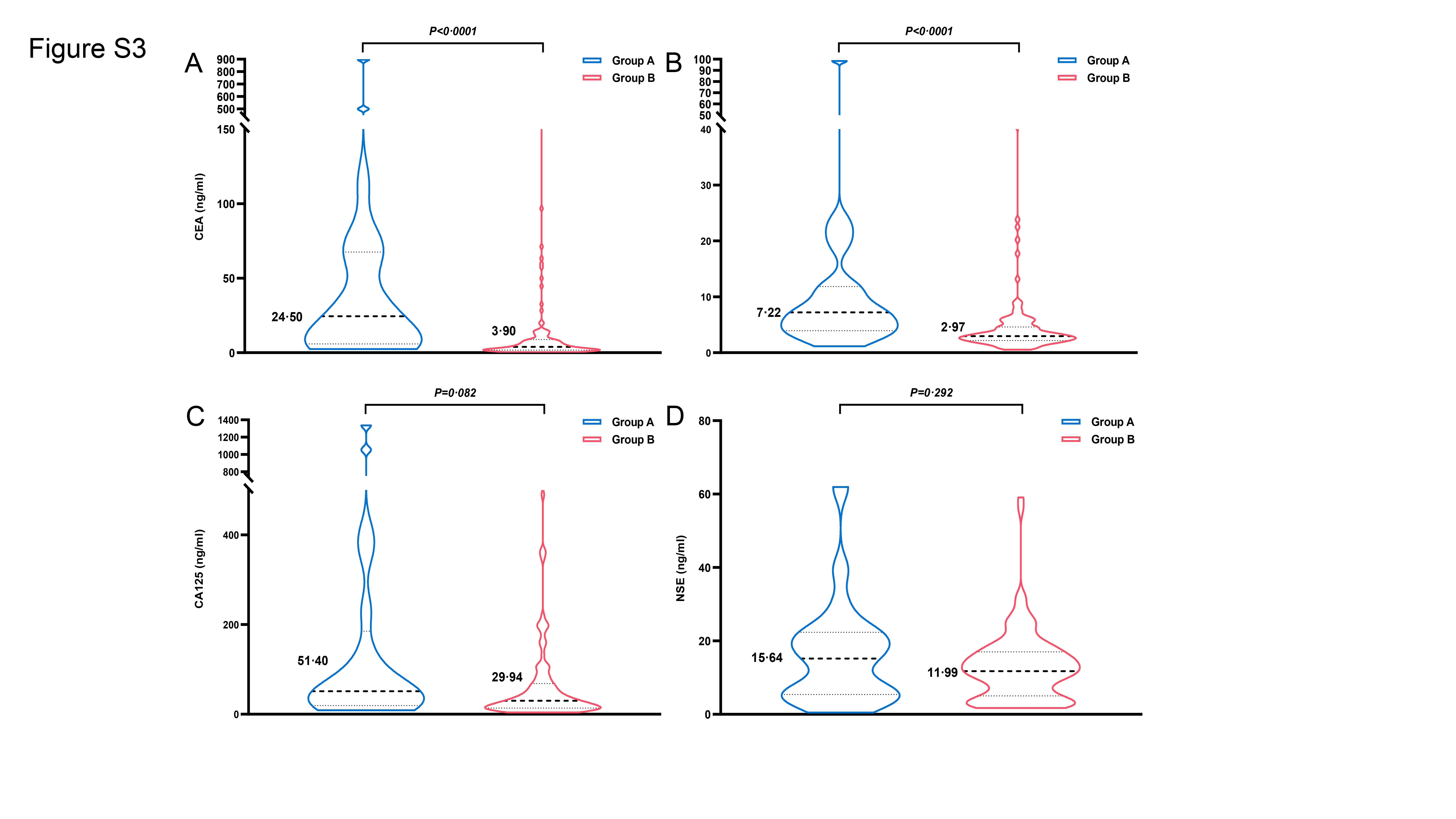

Supplement: Supplementary file 3 — Figure S3 [file CAM4-12-5603-s011.jpg]

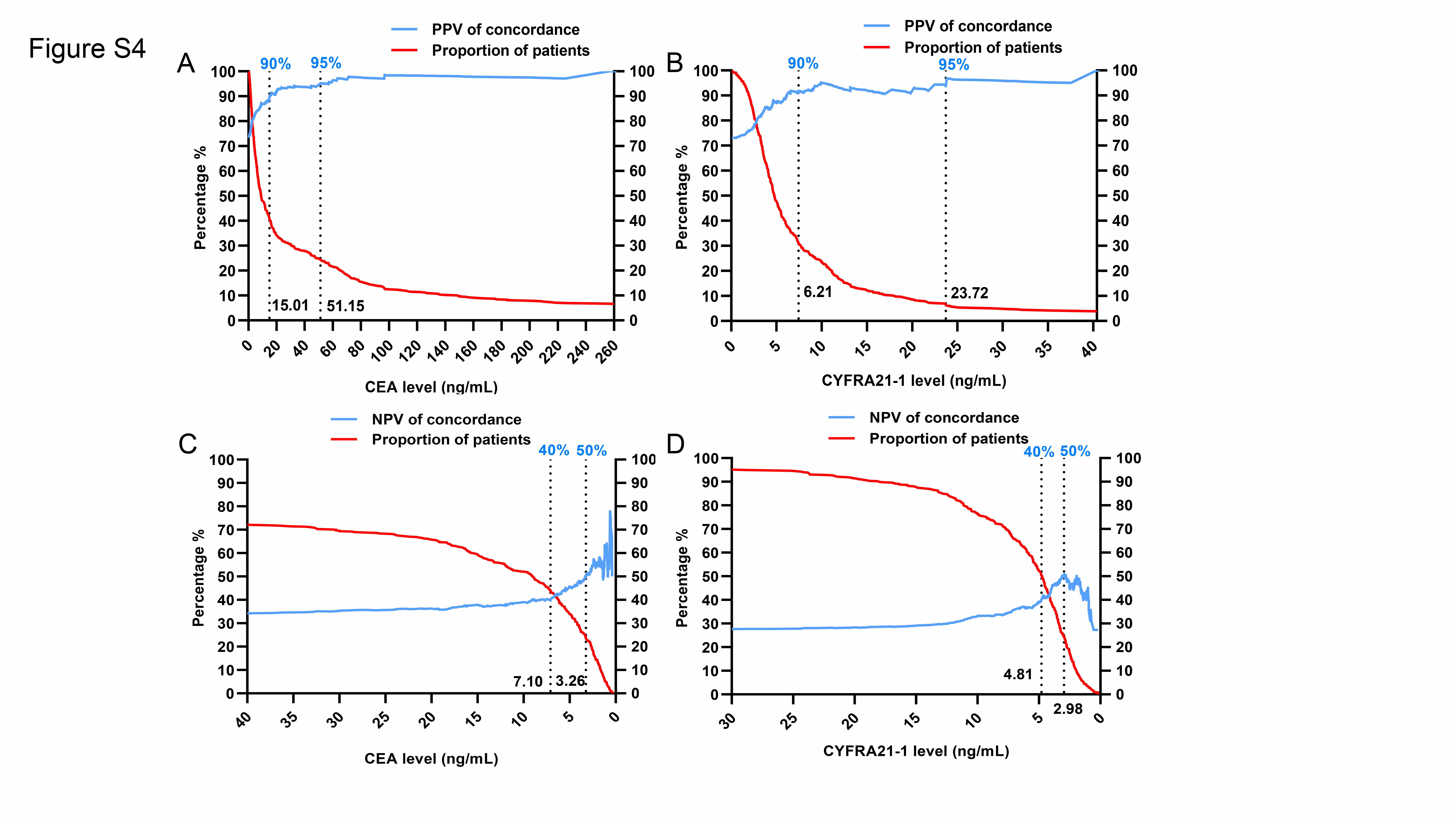

Supplement: Supplementary file 4 — Figure S4 [file CAM4-12-5603-s005.jpg]

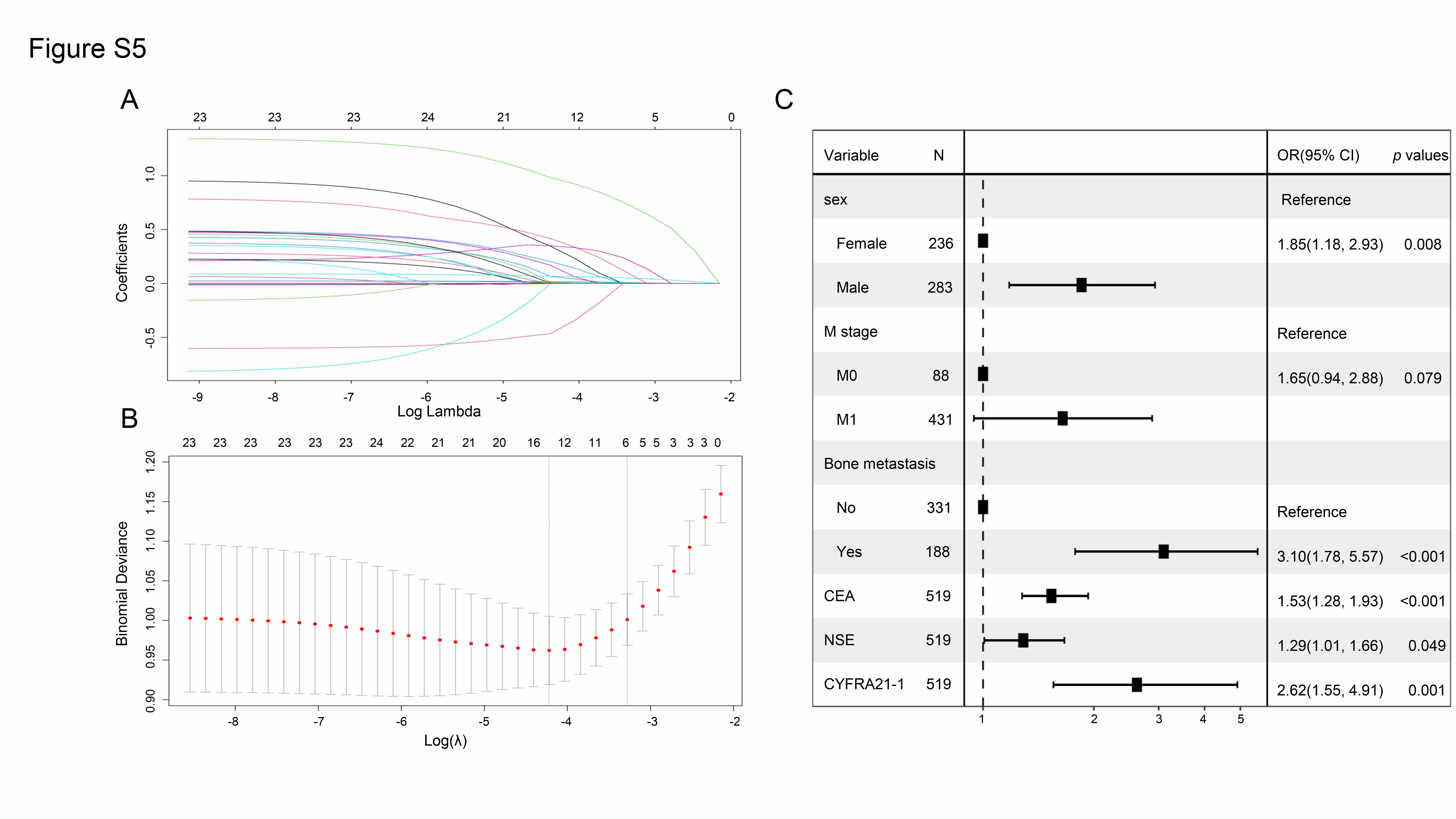

Supplement: Supplementary file 5 — Figure S5 [file CAM4-12-5603-s004.jpg]

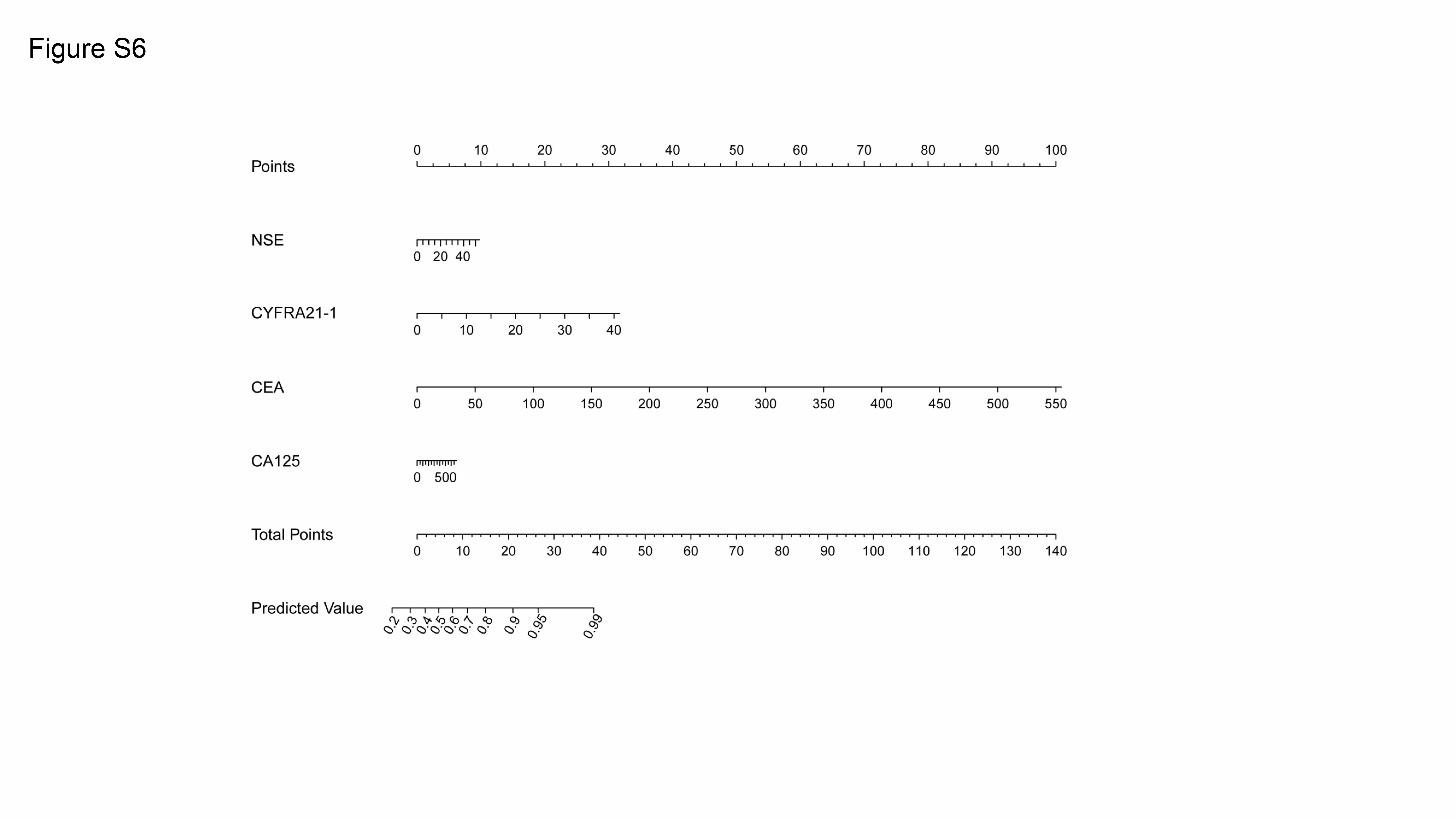

Supplement: Supplementary file 6 — Figure S6 [file CAM4-12-5603-s001.jpg]

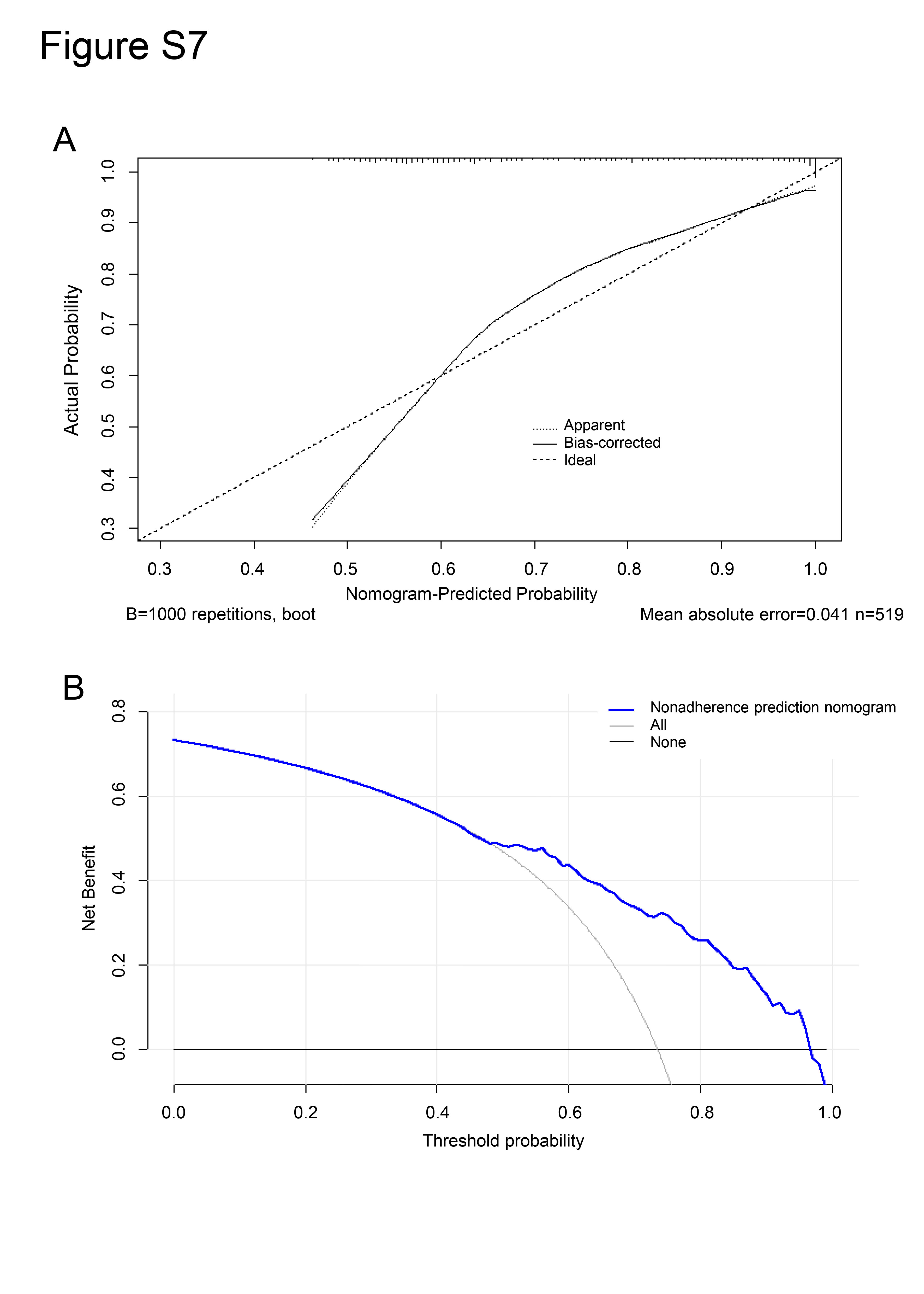

Supplement: Supplementary file 7 — Figure S7 [file CAM4-12-5603-s012.jpg]

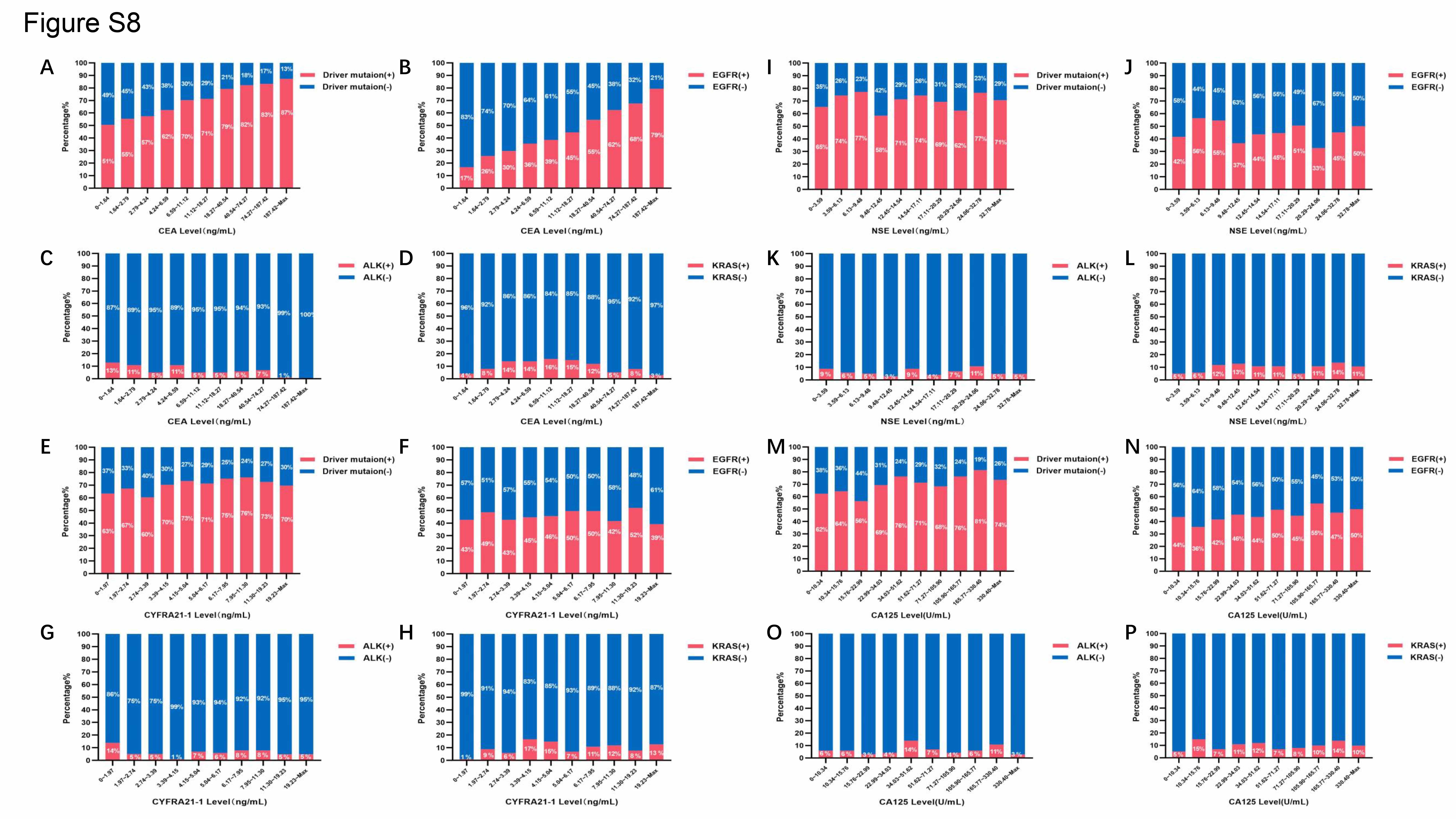

Supplement: Supplementary file 8 — Figure S8 [file CAM4-12-5603-s006.jpg]

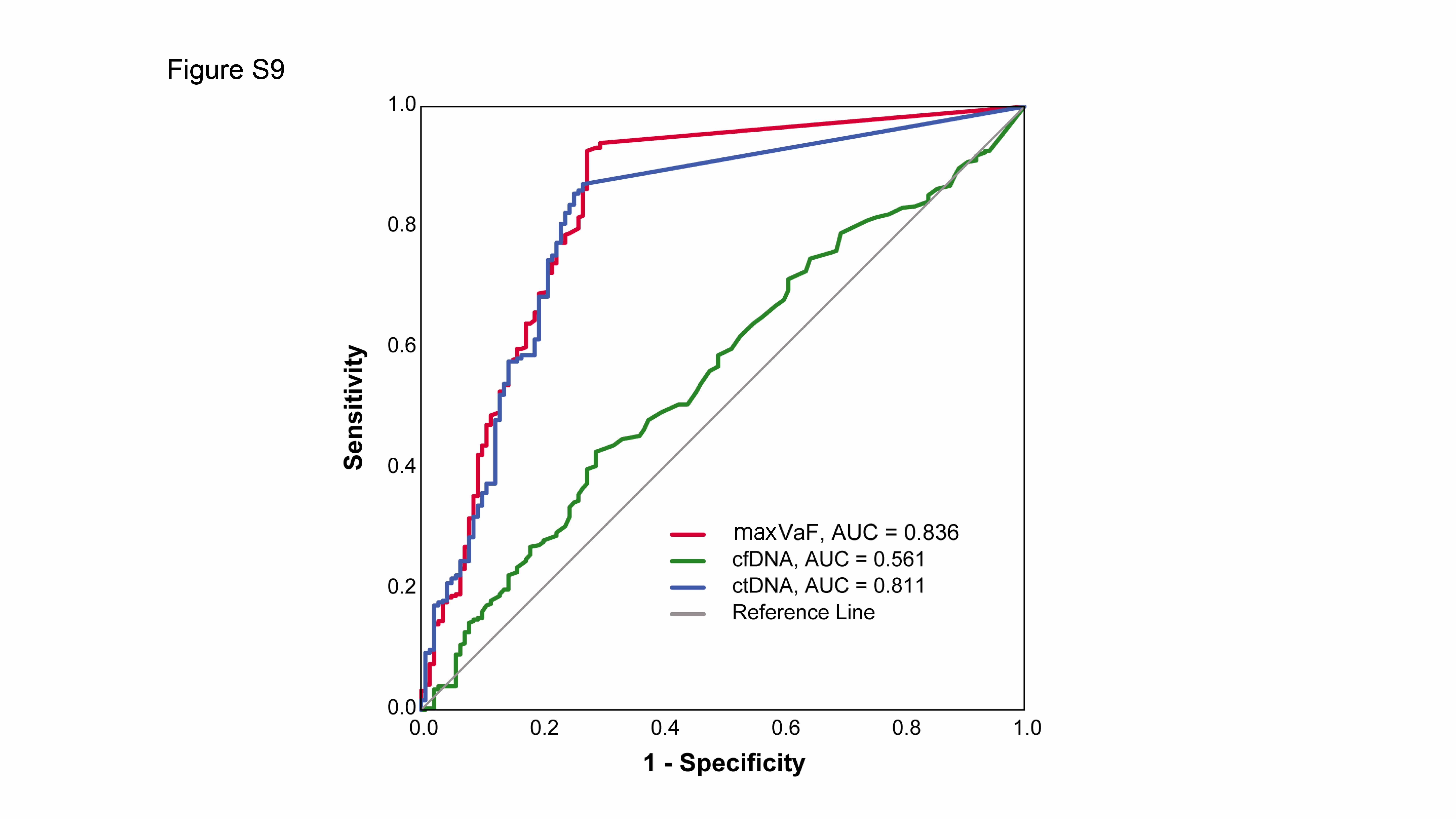

Supplement: Supplementary file 9 — Figure S9 [file CAM4-12-5603-s003.jpg]

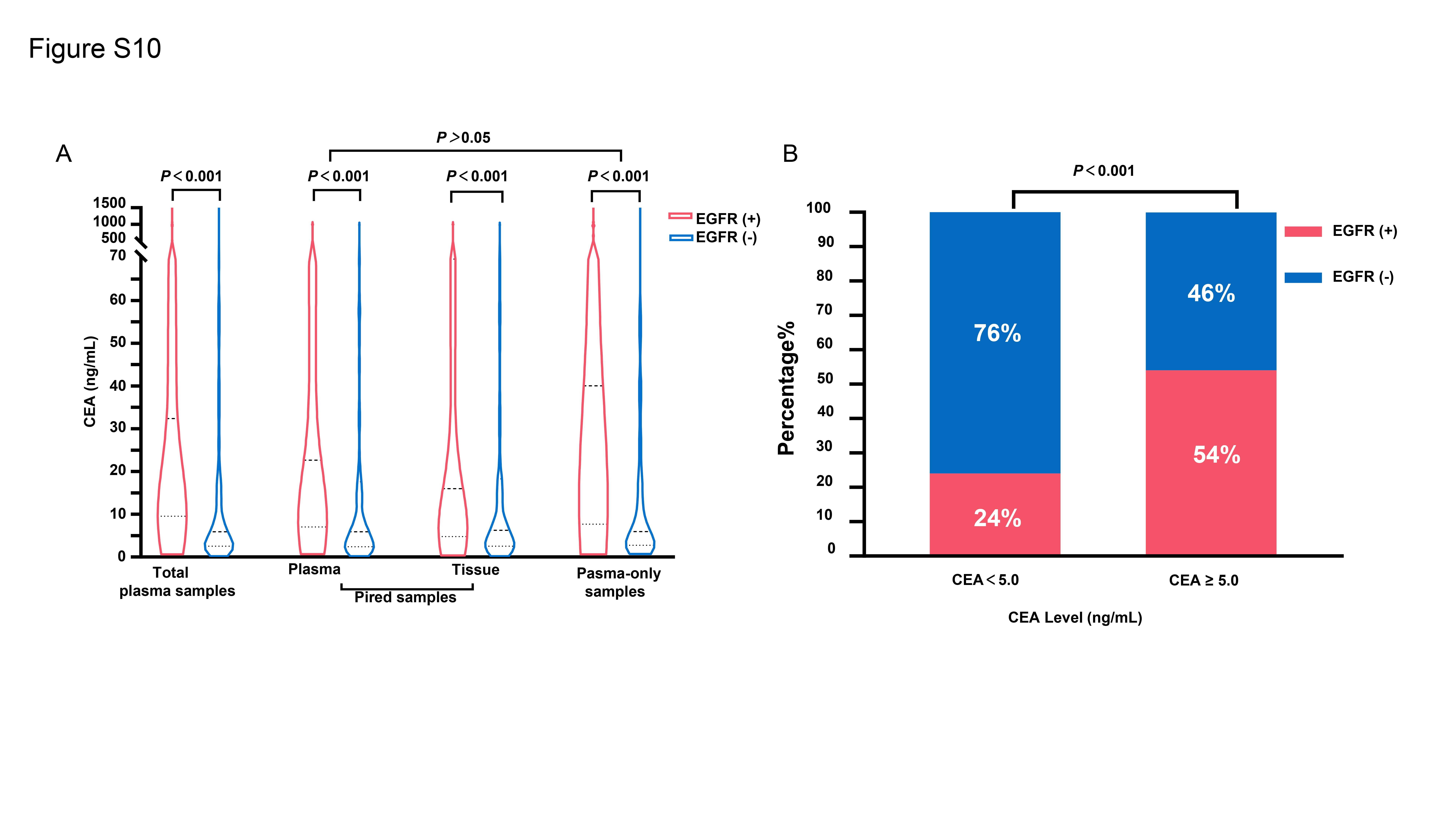

Supplement: Supplementary file 10 — Figure S10 [file CAM4-12-5603-s010.jpg]
