# Supplementary material for: The rational application of liquid biopsy based on next‐generation sequencing in advanced non‐small cell lung cancer
Source: Cancer Med. 2022 Nov 7;12(5):5603–14. doi: 10.1002/cam4.5410 (PMC10028052; doi:10.1002/cam4.5410)
Supplement: Supplementary file 11 — Appendix S1 [file CAM4-12-5603-s008.docx]

**Supplementary Methods.**

**1.1** **Preparation of plasma cfDNA**

10 ml of peripheral blood was collected into an anticoagulant tube and stored at 4℃, and then transferred to the centrifuge tube within 2h and centrifuged at 2,000 g for 10min at 4℃. The supernatant was transferred to a new 15mL centrifuge tube and centrifuged again at 16,000g for 10min at 4℃. The supernatant was transferred to a new 15ml centrifuge tube again and stored at -80°C for further processing. Cell-free DNA (cfDNA) was extracted using the QIAamp Circulating Acid Kit (Qiagen, Valencia, CA, USA) according to the manufacturer's instructions and its concentration was measured by Qubit 2.0 Fluorometer with dsDNA HS Detection Kit (Life Technologies, Carlsbad, CA, USA). A minimum of 20 ng of cfDNA was required for NGS library construction.

**1.2 Tumor tissue DNA extraction**

Tumor tissue samples were obtained by percutaneous puncture, bronchoscopy or thoracoscopy. Tissue DNA was extracted by QIAamp DNA FFPE Tissue Kit (Qiagen, Valencia, CA, USA) according to the manufacturer’s instructions. DNA concentration of tumor tissue was measured by Qubit dsDNA assay (Life Technologies, Carlsbad, CA, USA). A minimum of 50 ng of tumor tissue DNA was required for NGS library construction.

**1.3 NGS Library Preparation**

Covaris M220 is used for DNA shearing, followed by end repair, phosphorylation and adaptor ligation. Agencourt AMPure beads (Beckman Coulter, California, US) was used to select fragments of size 200–400bp, followed by hybridization with capture probes baits, hybrid selection with magnetic beads and PCR amplification. A bioanalyzer high-sensitivity DNA assay was used to size of the fragments and assess the quality**.** The indexed samples were sequenced on Nextseq500 sequencer (Illumina, Inc., California, US) with pair-end reads.

**1.4 Sequencing data analysis**

BWA aligner 0.7.10 was used to map sequencing data to the human genome (hg19). Local alignment optimization, annotation and variant calling were performed using GATK 3.2. Tophat2 and Factera 1.4.3 were used to DNA translocation analysis. Comparing plasma samples against their own white blood cells to identify somatic variants. The VarScan filter pipeline was used to filter variants and filter out loci with depth less than 100. The average sequencing depth of all target regions in tissue DNA was 2000×, and the average sequencing depth in cfDNA of plasma was 10,000×. According to 1000 Genomes Project, Exome Aggregation Consortium, ESP6500SI-V2, and dbSNP databases, variants with over 0.1% population frequency were classified as single-nucleotide polymorphisms (SNP) and excluded from further analysis. Remaining variants were annotated with the SnpEff v3.6 and ANNOVAR software.
