# Supplementary material for: The rational application of liquid biopsy based on next‐generation sequencing in advanced non‐small cell lung cancer
Source: Cancer Med. 2022 Nov 7;12(5):5603–14. doi: 10.1002/cam4.5410 (PMC10028052; doi:10.1002/cam4.5410)
Supplement: Supplementary file 12 — Table S1 Table S2 [file CAM4-12-5603-s007.docx]

Supplementary Table S1. Clinicopathological characteristics of patients

| **Characteristic** |  | **Total samples (*N*=1012)** | **Paired tissue and plasma samples (*N*=519)** | **Only plasma samples (*N*=493)** | ***P*** |
| --- | --- | --- | --- | --- | --- |
| Age(years), median (IQR) |  | 61 (53－67) | 60 (51－66) | 62 (54－69) | 0.002 |
| Gender, *n* (%) |  |  |  |  | 0.372 |
| Male |  | 538 (53.2) | 283 (54.5) | 255 (51.7) |  |
| Female |  | 474 (46.8) | 236 (45.5) | 238 (48.3) |  |
| Smoking status |  |  |  |  | 0.928 |
| Never |  | 607 (60.0) | 312 (60.1) | 295 (59.8) |  |
| Current or former |  | 405 (40.0) | 207 (39.9) | 198 (40.2) |  |
| T stage, *n* (%) |  |  |  |  | 0.002 |
| T1 |  | 142 (14.0) | 71 (13.7) | 71 (14.4) |  |
| T2 |  | 237 (23.4) | 126 (24.3) | 111 (22.5) |  |
| T3 |  | 140 (13.8) | 92 (17.7) | 48 (9.7) |  |
| T4 |  | 373 (36.9) | 170 (32.8) | 203 (41.2) |  |
| Unknown |  | 120 (11.9) | 60 (11.6) | 60 (12.2) |  |
| N stage, *n* (%) |  |  |  |  | 0.135 |
| N0 |  | 135 (13.3) | 68 (13.1) | 67 (13.6) |  |
| N1 |  | 73 (7.2) | 38 (7.3) | 35 (7.1) |  |
| N2 |  | 352 (34.9) | 182 (35.1) | 170 (34.5) |  |
| N3 |  | 418 (41.3) | 221 (42.6) | 197 (40.0) |  |
| Unknown |  | 34 (3.4) | 10 (1.9) | 24 (4.9) |  |
| M stage, *n* (%) |  |  |  |  | 0.062 |
| M0 |  | 151 (14.9) | 88 (17.0) | 63 (12.8) |  |
| M1 |  | 861 (85.1) | 431 (83.0) | 430 (87.2) |  |
| Stage, *n* (%) |  |  |  |  | 0.062 |
| III |  | 151 (14.9) | 88 (17.0) | 63 (12.8) |  |
| IV |  | 861 (85.1) | 431 (83.0) | 430 (87.2) |  |
| Metastatic site, *n* (%) |  |  |  |  | 0.759 |
| Contralateral lung |  | 358 (35.4) | 179 (34.5) | 179 (36.3) |  |
| Pleura |  | 406 (40.1) | 187 (36.0) | 219 (44.4) |  |
| Bone |  | 396 (39.1) | 188 (36.2) | 208 (42.2) |  |
| Brain |  | 206 (20.4) | 103 (19.9) | 103 (20.9) |  |
| Liver |  | 114 (11.3) | 54 (10.0) | 60 (12.2) |  |
| Pericardium |  | 88 (8.7) | 50 (9.6) | 38 (7.7) |  |
| Adrenal gland |  | 104 (10.3) | 51 (9.8) | 53 (10.8) |  |
| Others |  | 40 (4.0) | 20 (3.9) | 20 (4.1) |  |
| Therapy, *n* (%) |  |  |  |  | ＜0.001 |
| 1st line |  | 797 (78.8) | 490 (94.0) | 307 (62.3) |  |
| ≥2nd line |  | 215 (21.2) | 29 (6.0) | 186 (37.7) |  |

Supplementary Table S2. Driver mutations and Variation type

| **Driver mutations** | **Variation type** |
| --- | --- |
| EGFR | L858R,19del,G719A/S,E709A/K,T790M,C797S,20ins,L861Q |
| ALK | Fusion |
| ROS1 | Fusion |
| RET | Fusion |
| MET | Exon14 skip, Amplification |
| BRAF | V600E |
| ERBB2 | Exon20 insertion, Amplification |
| NTRK | Fusion |
